# Phase II randomized controlled trial comparing traditional Thai cannabis-based medicine with lorazepam for insomnia treatment

**DOI:** 10.1186/s42238-026-00415-x

**Published:** 2026-02-24

**Authors:** Thavatchai Kamoltham, Suwadee Chokchaisiri, Chawalit Yongram, Panupan Sripan, Surasak Im-iam, Panupong Sanasit, Varanon Intaravattana, Chatchai Sawasdichai, Patpong Udompat, Tanawat Chaiphongpachara, Tanawan Kummalue

**Affiliations:** 1 Department of Cannabis Health Sciences, College of Allied Health Sciences, Suan Sunandha Rajabhat University, Samut Songkhram, 75000 Thailand; 2 Department of Thai Traditional and Alternative Medicine, Prapokklao Hospital, Chanthaburi, 22000 Thailand; 3 Department of Public Health and Health Promotion, College of Allied Health Sciences, Suan Sunandha Rajabhat University, Samut Songkhram, 75000 Thailand; 4 Faculty of Medicine, Siriraj Hospital, Mahidol University, Bangkok, 10700 Thailand

**Keywords:** Thai Herbal Medicine, Medical Cannabis, Insomnia Treatment, Anti-Pom-Leung Fever Medicine, Complementary and Alternative Medicine

## Abstract

**Background:**

A traditional Thai cannabis-based multi-herbal formulation is legally recognized in Thailand for therapeutic use and clinical research. However, clinical evidence supporting its use for insomnia remains limited.

**Methods:**

This Phase II randomized, double-blind, active-controlled non-inferiority trial compared the efficacy and safety of the Anti-Pom-Leung Fever medicine with lorazepam in patients with chronic insomnia. One hundred participants were randomized to receive either the herbal formulation or lorazepam for 4 weeks. Sleep quality was assessed using the Pittsburgh Sleep Quality Index (PSQI). Non-inferiority was evaluated at week 4 using a predefined margin of 2.1 based on the upper bound of the two-sided 95% confidence interval for the mean PSQI difference (experimental minus comparator). Longitudinal changes were analyzed using repeated-measures analysis of variance, and safety was evaluated through clinical and laboratory assessments.

**Results:**

Eighty-two participants completed the study (41 per group). Baseline characteristics were comparable between groups. Both treatments significantly improved sleep quality over 4 weeks. At week 4, mean PSQI scores were 3.44 in the experimental group and 4.78 in the comparator group, with a mean difference of -1.34 (95% CI: -2.99 to 0.31), demonstrating non-inferiority. A significant main effect of time on PSQI scores was observed, with no significant time-by-treatment interaction. Quality of life and stress improved in both groups, and safety profiles were comparable, with no clinically significant adverse effects.

**Conclusions:**

The traditional Thai cannabis-based multi-herbal formulation demonstrated non-inferior efficacy to lorazepam and was well tolerated, supporting its use as a short-term alternative for chronic insomnia.

## Introduction

Insomnia is a pervasive global public health concern, with recent reports indicating a prevalence of up to 40% in the Thai population (Leewongcharoen 2024; Roth 2007). Chronic insomnia significantly impairs daily functioning and quality of life, necessitating effective therapeutic interventions. Current pharmacological treatments often rely on benzodiazepines, such as lorazepam, which are effective in improving sleep latency and duration but are associated with risks of dependence and adverse side effects (Minkel and Krystal 2013; Ghiasi et al. 2024). This has driven interest in exploring well-tolerated, naturally derived alternatives.

In Thailand, a unique regulatory environment has facilitated the study of traditional medicinal formulas. The Anti-Pom-Leung Fever medicine, also known as the Cannabis mixture (Prasa Kuncha), is a time-honored formula recognized under Thai law for therapeutic use, including the treatment of insomnia. Its historical use is documented in the original Royal Thai Medical Textbook, *Patsart Song Klor*, dating back more than 150 years to the reign of King Rama V. This formula is a complex herbal remedy officially listed in the National Thai Traditional Medicine Pharmacopeia (Department of Medical Sciences 2021).

The traditional Thai cannabis-based medicine consists of eight herbal components combined in a specific ratio: dry ginger (1 part), ground pepper (2 parts), Dee-pli (long pepper, 1 part), red sandalwood (1 part), white sandalwood (1 part), neem leaf (1 part), five-leaved chaste leaf (1 part), and notably *Cannabis sativa* leaves (8 parts), which form the major component by weight (50% of the formulation) (Phraya Phitsanuphrasatwet 1909). Although the medicine was historically documented for treating “Pom-Leung Fever” (a traditional designation for conditions involving insomnia and wasting syndrome), its use in traditional clinical practice has broadened to include symptom management for associated conditions, particularly sleep disturbance and anxiety. Given the growing body of evidence supporting the role of phytocannabinoids in sleep improvement due to their favorable tolerability profile (Walsh et al. 2021), the official incorporation of cannabis into this traditional remedy represents a culturally significant and scientifically relevant therapeutic pathway. Traditional formulas containing cannabis are domestically recognized for the treatment of insomnia (Jamparngernthaweesri et al. 2023).

The extensive history of traditional use and the formula’s inclusion in the National Thai Traditional Medicine Pharmacopeia establish its preliminary safety profile, consistent with a reverse-pharmacology approach that allows clinical investigation to bypass a formal Phase I trial for new chemical entities (Patwardhan 2009). Accordingly, no formal Phase I clinical trial was conducted for this formulation. Consequently, the present research was designed as a Phase II trial to generate definitive efficacy and safety data compared with standard treatment, in alignment with regulatory pathways for complex traditional medicines transitioning toward evidence-based practice. Despite historical recognition and legal approval, robust contemporary clinical data establishing the efficacy of this traditional cannabis-based medicine relative to modern conventional treatments remain limited. To address this gap, the study first aimed to chemically characterize and quantify the cannabinoid composition of the traditional Thai cannabis-based medicine. Therefore, a Phase II randomized, active comparator-controlled outpatient clinical trial was conducted to determine its efficacy and safety, in terms of sleep quality, stress levels, and quality of life, compared directly with lorazepam, a commonly used intermediate-acting treatment for chronic insomnia in Thailand.

## Materials and methods

### Ethical approval and study design

This study was a Phase II, randomized, double-blind, active-controlled trial conducted at the Department of Thai Traditional and Alternative Medicine, Prapokklao Hospital, Chanthaburi, Thailand, from August 2023 to February 2024. The study was designed as a non-inferiority trial to test the hypothesis that the traditional herbal remedy is not inferior in efficacy to lorazepam for the treatment of chronic insomnia. The study was conducted in accordance with the Declaration of Helsinki and adhered to Good Clinical Practice (GCP) guidelines. The protocol was reviewed and approved by the Ethics Committee for Research in Human Subjects, Department of Thai Traditional and Alternative Medicine (approved February 6, 2023; Protocol ID: 06-2565), and the Chanthaburi Provincial Public Health Office/Health Region 6 Ethics Committee (approved July 21, 2023; Protocol ID: 036/66). All participants provided written informed consent prior to enrollment.

### Participant recruitment and screening

Participants were recruited through hospital bulletins and social media advertisements targeting outpatients of Prapokklao Hospital and residents of Chanthaburi Province, Thailand. Interested individuals underwent a multi-stage screening process. Initial screening included a clinical interview and physical examination to obtain medical history and assess general health status. Sleep disturbance was screened using the Pittsburgh Sleep Quality Index (PSQI). Baseline clinical laboratory investigations, including complete blood count and liver and kidney function tests, were performed to confirm medical eligibility prior to enrollment. Individuals who met all eligibility criteria were provided with a detailed explanation of the study procedures by study personnel or research nurses. Eligible participants were then asked to provide written informed consent by signing the informed consent form prior to participation in the study.

### Eligibility criteria

#### Inclusion criteria

1. Thai men or women aged 25 to 70 years.
2. Presence of insomnia symptoms for at least 1 month, with a Pittsburgh Sleep Quality Index (PSQI) score greater than 5.
3. Adequate baseline laboratory results prior to study enrollment, including an estimated glomerular filtration rate (eGFR) greater than 60 mL/min, aspartate aminotransferase (AST) and alanine aminotransferase (ALT) levels less than three times the upper limit of normal, and a hemoglobin level (Hb) greater than 10 g/dL.
4. Female participants of childbearing potential were required to have a negative urine pregnancy test at screening.
5. Willingness to participate voluntarily in the study and to provide written informed consent prior to enrollment.

#### Exclusion criteria

1. Patients with hepatic or renal abnormalities.
2. Patients with cardiovascular disease, including hypertension or gastroesophageal reflux disease (GERD), or those using anticoagulant medications (including warfarin, aspirin, or clopidogrel), as well as antihypertensive medications.
3. Patients using phenytoin, propranolol, theophylline, or rifampicin.
4. Individuals currently using other herbal medicines or those with a history of allergic reactions to herbal medicines.
5. Individuals who consumed alcohol within 1 month prior to study enrollment or during the study period.
6. Individuals with a history of illicit drug use or substance abuse.
7. Pregnant or breastfeeding women.

### Preparation of investigational products

#### Anti-Pom-Leung Fever medicine

All herbal ingredients used in the preparation of the Anti-Pom-Leung Fever medicine were processed in accordance with standardized traditional Thai medicine procedures to ensure consistency and quality (Department of Medical Sciences 2021). The composition of the Anti-Pom-Leung Fever medicine is shown in Table 1. Cannabis leaves were sourced from the verified “Hang Kra Rog Phu Phan” indigenous Thai strain, with identification confirmed through phenotypic, microscopic, chemical, and genotypic analyses (Kamoltham et al. 2025). All herbal components underwent rigorous testing to ensure that they were free from heavy metals, insecticides, fungi, bacteria, and yeast.

**Table 1:** Composition of the Anti-Pom-Leung Fever Medicine

No.	Common name	Scientific name	Plant part used	Amount (g)
1	Dried ginger	*Zingiber officinale*	Rhizome	1
2	Black pepper	*Piper nigrum*	Seed	2
3	Indian long pepper	*Piper retrofractum*	Fruit spike	1
4	Red sandalwood	*Dracaena cochinchinensis*	Heartwood	1
5	White sandalwood	*Santalum album*	Heartwood	1
6	Neem	*Azadirachta indica*	Leaf	1
7	Five-leaved chaste tree	*Vitex negundo*	Leaf	1
8	Cannabis	*Cannabis sativa*	Leaf	8

Briefly, in the preparation of the Anti-Pom-Leung Fever medicine, crude herbal materials were initially cleaned to remove visible contaminants. Cannabis leaves were prepared separately using a traditional roasting (satu) process: the leaves were washed three times with clean water, drained, lightly roasted over low heat for 3–5 min until dry and brittle, allowed to cool, and then stored in airtight containers. All other herbal ingredients were dried in a hot-air oven at 50–55 °C for 4–6 h, or until completely dry. The dried components were weighed according to the prescribed formulation ratios, finely ground, sieved through an 80-mesh sieve, and thoroughly mixed to ensure homogeneity. The final blended powder was encapsulated into hard gelatin capsules with a fill weight of 500 mg per capsule.

Quality control of the raw herbal materials used in the Anti-Pom-Leung Fever medicine was conducted in accordance with the Thai Herbal Pharmacopoeia (Department of Medical Sciences 2021). The procedures included macroscopic examination to verify the external characteristics of each crude drug, followed by microscopic identification of diagnostic tissue features using appropriate reagents and staining techniques. Foreign matter was assessed by visual separation and expressed as a percentage of the total sample weight. Physicochemical evaluations included the determination of total ash, acid-insoluble ash, loss on drying, and ethanol- and water-soluble extractive values using standardized pharmacopeial methods. In addition, volatile oil content was quantified by hydrodistillation. All analyses were performed to ensure the identity, purity, and quality consistency of the herbal raw materials prior to formulation.

#### Lorazepam (active comparator)

Lorazepam capsules were prepared as the active comparator using a compounding process to ensure dose accuracy and maintain blinding. Lorazepam (0.5 mg) tablets were finely triturated and mixed with pharmaceutical-grade lactose, then encapsulated into hard gelatin capsules with a total fill weight comparable to that of the herbal capsules. For the placebo capsules, lactose was used as the sole excipient, constituting 100% w/w of the formulation. Each placebo capsule contained 367 mg of lactose, corresponding to 3,670 g for the production of 10,000 capsules. For the lorazepam capsules, the formulation consisted of lactose (86.95% w/w; 333 mg per capsule) and lorazepam tablet powder (13.05% w/w; 50 mg per capsule), yielding a total capsule weight of 383 mg. This corresponded to 500 g of lactose and 75 g of lorazepam tablet powder for the preparation of 1,500 capsules. The amount of lorazepam tablet powder per capsule was calculated to deliver an equivalent dose of 0.5 mg lorazepam per capsule.

The placebo capsules were evaluated for physical quality in accordance with USP 40 (United States Pharmacopeial Convention 2017a, b). Weight variation was assessed by weighing 20 capsules individually, with acceptable limits defined as 90–110% of the mean weight. If any capsule fell outside this range, net fill weights were determined and additional testing was performed as specified by pharmacopeial criteria. Disintegration testing was conducted using a standard disintegration apparatus, with six capsules tested in water maintained at 37 ± 2 °C. Capsules were considered compliant if complete disintegration occurred within 30 min, with confirmatory testing performed when necessary in accordance with USP requirements. All procedures were conducted at the Department of Pharmacology, Mahidol University, Thailand.

### Analysis of cannabinoids in Anti-Pom-Leung Fever medicine

To characterize the cannabinoid profile of the Anti-Pom-Leung Fever medicine, extraction and quantification were performed using high-performance liquid chromatography (HPLC). This technique was employed for the extraction and quantification of cannabinoids due to its high accuracy, sensitivity, and reproducibility, and it is widely recognized as a standard method for cannabinoid analysis (Herrera et al. 2024).

#### Extraction procedure

A 200 mg sample of the medicine was weighed into a 50 mL centrifuge tube. Extraction was initiated by adding 25 mL of 80% aqueous methanol. The mixture was agitated using a vortex mixer for 30 s and subsequently submerged in an ultrasonic bath for 15 min. After extraction, 100 µL of the supernatant was transferred to a 1.5 mL microcentrifuge tube and diluted with 900 µL of 80% aqueous methanol. The diluted solution was vortexed for 30 s and filtered through a 0.22 μm PTFE syringe filter into a 1.5 mL HPLC vial.

#### HPLC

Analysis was conducted using a Shimadzu Prominence-i LC-2030 C 3D HPLC system equipped with a Shimadzu NexLeaf CBX for potency C18 column (150 × 4.6 mm, 2.7 μm particle size; Columbia, MD, USA). The mobile phase used a gradient system comprising 0.085% H₃PO₄ in water (Solvent A) and 0.085% H₃PO₄ in acetonitrile (Solvent B). The flow rate was maintained at 1.6 mL/min, and the column temperature was 35 °C. A 5 µL sample volume was injected. Detection was performed using a UV diode-array detector at 220 nm.

#### Quantification

The concentrations of ten cannabinoids, including CBDV, CBDA, CBGA, CBG, CBD, THCV, CBN, Δ⁹-THC, CBC, and THCA, were quantified by peak area integration relative to certified reference standards (Sigma-Aldrich, USA).

### Randomization and blinding

A total of 100 eligible participants were randomized in a 1:1 ratio to two treatment arms using a computer-generated block randomization schedule (nQuery Advisor; block size 4). To maintain the double-blind design, medications were provided in identical, opaque capsules.

#### Blinding protocol

To prevent identification of the active agents, a four-code system (A, B, C, and D) was used. Codes A and B were assigned to the experimental cannabis formula, while codes C and D were assigned to the lorazepam/placebo combinations. An independent pharmacist managed the coding and dispensing. Neither the clinical investigators nor the participants were aware of group assignments until the final dataset was locked for analysis.

#### Intervention and administration

Study medications were administered for 28 consecutive days using a double-dummy technique to maintain blinding. Participants were randomly assigned to one of two groups: a comparator group receiving lorazepam and an experimental group receiving the Anti-Pom-Leung Fever medicine. Participants in both groups were provided with take-home study medications using equivalent dosing regimens, and the medications were identical in appearance, ensuring that neither the participants nor the investigators could distinguish between treatment assignments.

Participants in the experimental (trial) group received Anti-Pom-Leung Fever medicine capsules (500 mg per capsule) administered twice daily. They received four capsules before breakfast and four capsules at bedtime, representing the standard therapeutic dosage of this formulation for the treatment of insomnia in Thailand. Participants in the comparator group received matching placebo capsules to maintain an identical capsule count and dosing schedule. Specifically, they received four placebo capsules before breakfast. At bedtime, they received one capsule containing lorazepam 0.5 mg together with three placebo capsules, resulting in a total of four capsules administered at bedtime. The lorazepam dose of 0.5 mg once daily at bedtime was selected in accordance with standard medical practice recommendations in Thailand for the treatment of insomnia (Department of Thai Traditional and Alternative Medicine 2025).

After enrollment, participants in all groups were provided with a sleep quality assessment questionnaire to take home within 1 week. The questionnaire was used to facilitate responses during telephone follow-up calls conducted by study staff or research nurses. Participants were contacted by telephone to monitor symptoms and assess sleep quality on Day 7 (Week 1), Day 14 (Week 2), and Day 21 (Week 3) after initiating the study medication.

Participants were subsequently scheduled for an in-person follow-up visit at Week 4 (Day 28) after enrollment and treatment initiation. Prior to this visit, participants were instructed to fast for at least 12 h for blood sample collection. On the appointment day, participants were required to return any remaining study medication. Blood samples were collected, and electrocardiography (ECG) was performed. Participants also underwent a medical history review and physical examination, and completed assessments of sleep quality, quality of life, pain, and perceived stress. Blood samples were collected from participants in both groups on two occasions, at baseline (Day 0) and on Day 28, to evaluate laboratory blood parameters.

### Outcome measures

#### Clinical laboratory and safety assessments

To monitor safety and physiological impact, comprehensive clinical laboratory tests were performed at baseline and upon treatment completion. These included liver function (AST, ALT, ALP), renal function (serum creatinine and blood urea nitrogen), pancreatic function, and lipid profiles (total cholesterol, triglycerides, HDL, and LDL). Fasting blood sugar and hematological parameters (white blood cell count, hemoglobin, and platelet count) were also recorded. Cardiac safety was monitored via electrocardiograms (EKG).

#### Sleep quality

The primary efficacy endpoint was sleep quality measured by the Pittsburgh Sleep Quality Index (PSQI; Buysse et al. 1989). Assessments were conducted weekly for four weeks using standardized case report forms. PSQI scores range from 0 to 21, with scores > 5 indicating clinical insomnia.

#### Quality of life

Quality of life was assessed using the Pictorial Thai Quality of Life questionnaire (PTQL), a culturally adapted, self-administered instrument developed and validated for use in Thai populations (Phattharayuttawat et al. 2005). The PTQL consists of 25 pictorial items covering six domains: physical, cognitive, affective, social functioning, economic status, and self-esteem. Each item is presented in a pictorial format with ordinal response options reflecting symptom frequency or severity, allowing reliable self-assessment across a wide range of educational and literacy levels. Total scores range from 0 to 72 and were categorized as poor (0–24), moderate (25–49), or good (50–72) quality of life.

#### Stress levels

Perceived stress was assessed using the Stress Test-5 (ST-5), a standardized self-report screening tool developed by the Department of Mental Health, Ministry of Public Health, Thailand (Department of Mental Health 2023). The ST-5 consists of five items evaluating stress-related symptoms experienced over the preceding 2–4 weeks, including sleep disturbance, reduced concentration, irritability or restlessness, feelings of boredom or low mood, and social withdrawal. Each item is rated on a 4-point Likert scale ranging from 0 (rarely or not at all) to 3 (regularly), yielding a total score range of 0–15, with higher scores indicating greater levels of perceived stress. Stress severity was categorized according to the official scoring criteria as follows: low stress (0–4 points), moderate stress (5–7 points), high stress (8–9 points), and very high stress (10–15 points).

### Statistical analysis

#### Sample size and power analysis

The primary outcome measure for the power analysis was the change in PSQI score from baseline to week 4. In the absence of direct pharmacological comparative trials using PSQI in a similar setting, assumptions regarding the expected magnitude of PSQI improvement were informed by a published institutional report from the Institute of Geriatric Medicine, Department of Medical Services, Ministry of Public Health (Thailand), which evaluated a behavioral sleep intervention in older adults and observed a mean PSQI change from 8.27 ± 2.15 to 2.90 ± 1.04 (Institute of Geriatric Medicine 2008). This report involved a small elderly population and was not a randomized controlled trial; therefore, it was used only to provide a general reference for the potential scale of PSQI change rather than as a directly comparable clinical estimate. To account for uncertainty and population differences, the standard deviation (SD) was conservatively set at 3.2 points (1.5 × the reported SD of 2.15). The non-inferiority margin (δ) was defined as 2.1 points, corresponding to 10% of the maximum PSQI score (21 points) and considered clinically acceptable for sleep-quality outcomes. Using nQuery software with a significance level (α) of 0.05 and 90% power, the required sample size was 41 participants per group (82 total). Allowing for an anticipated 15% dropout rate, the target sample size was increased to 97. Ultimately, 100 participants were enrolled to ensure adequate statistical power.

#### Data analysis

Descriptive statistics were used to summarize continuous variables (mean ± SD or median and interquartile range), while categorical variables (e.g., sex) were presented as frequencies and percentages. Data distributions were assessed using normality tests. Continuous variables were compared between groups using an independent t-test or the Mann–Whitney U-test, as appropriate. Categorical data were analyzed using the chi-square test or Fisher’s exact test, with 95% confidence intervals (CIs) calculated for mean differences.

Non-inferiority was assessed at week 4 using the mean difference in PSQI scores (experimental minus comparator). Non-inferiority was concluded if the upper bound of the two-sided 95% confidence interval for the between-group difference was less than the prespecified non-inferiority margin (Δ = 2.1), corresponding to a one-sided α of 0.025. Longitudinal changes in PSQI, QOL, and stress scores over the four-week period were evaluated using repeated-measures analysis of variance (ANOVA) to assess time-by-treatment interactions. Statistical significance for all tests was set at *p* < 0.05.

## Results

### Participant flow and baseline characteristics

**Fig. 1:**
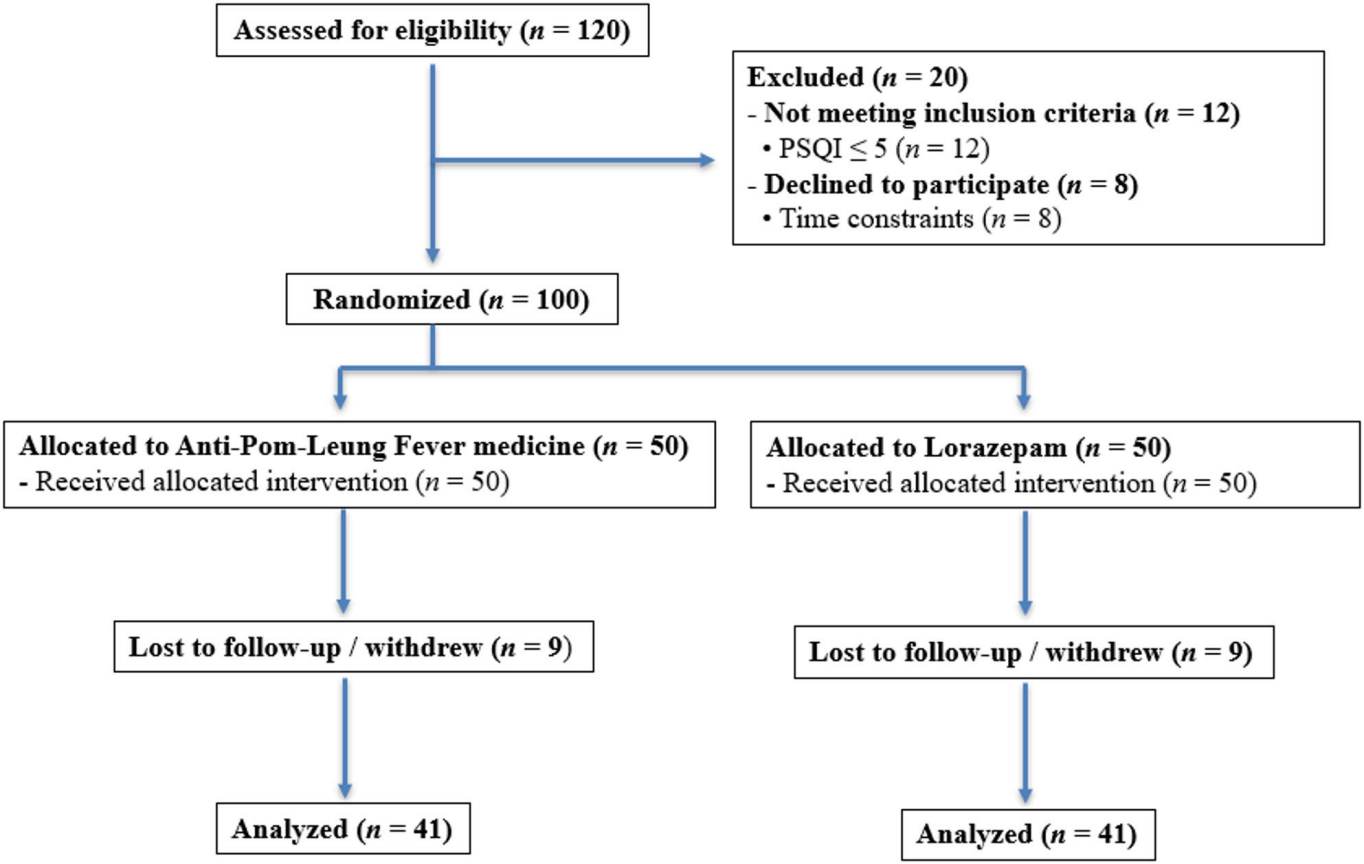
CONSORT flow diagram showing participant inclusion, randomization, and progression throughout the study

A total of 120 individuals were screened for eligibility. Of these, 20 were excluded: 12 did not meet the inclusion criteria (primarily PSQI ≤ 5), and 8 declined to participate due to time constraints. The remaining 100 participants were randomized into the experimental Anti-Pom-Leung Fever medicine group (*n* = 50) or the lorazepam comparator group (*n* = 50). During the four-week treatment period, 9 participants in the experimental group and 9 participants in the comparator group withdrew or were lost to follow-up, leaving 41 participants per group who completed the study (Fig. 1).

Baseline characteristics were well balanced between the experimental and comparator groups (Table 2). There were no significant differences in mean age (47.02 vs. 46.66 years; *p* > 0.05) or sex distribution (7/34 vs. 5/36 male/female; *p* > 0.05). Baseline body mass index (BMI) was also comparable between the two groups (24.95 vs. 23.41 kg/m²; *p* > 0.05). Similarly, baseline outcome measures, including PSQI, QOL, and stress levels, showed no statistically significant differences between the groups (*p* > 0.05).

**Table 2:** Baseline characteristics of the experimental and comparator groups

Parameter	Experimental group	Comparator group	*p*-value
*Demographic characteristics*			
Avg. age	47.02 ± 9.50	46.66 ± 11.05	0.873
Sex (M/F)	7/34	5/36	0.532
BMI	24.95 ± 4.36	23.41 ± 3.31	0.075
*Baseline outcome measures*			
Mean PSQI (Sleep Quality)	12.44 ± 2.31	13.05 ± 2.65	0.270
Mean QOL (Quality of Life)	53.71 ± 10.21	49.90 ± 13.83	0.160
Mean Stress	4.39 ± 2.44	4.90 ± 2.61	0.361

*Abbreviations: BMI* body mass index, *PSQI* Pittsburgh Sleep Quality Index, *QOL* quality of life. Stress was assessed using the Stress Test-5(ST-5). Data are presented as mean ± standard deviation (SD) unless otherwise indicated. Intergroup comparisons were performed using the independent-samples t-test for continuous variables, while categorical variables were compared using the chi-square test. A *p* value < 0.05 was considered statistically significant

### Cannabinoid profile

**Fig. 2:**
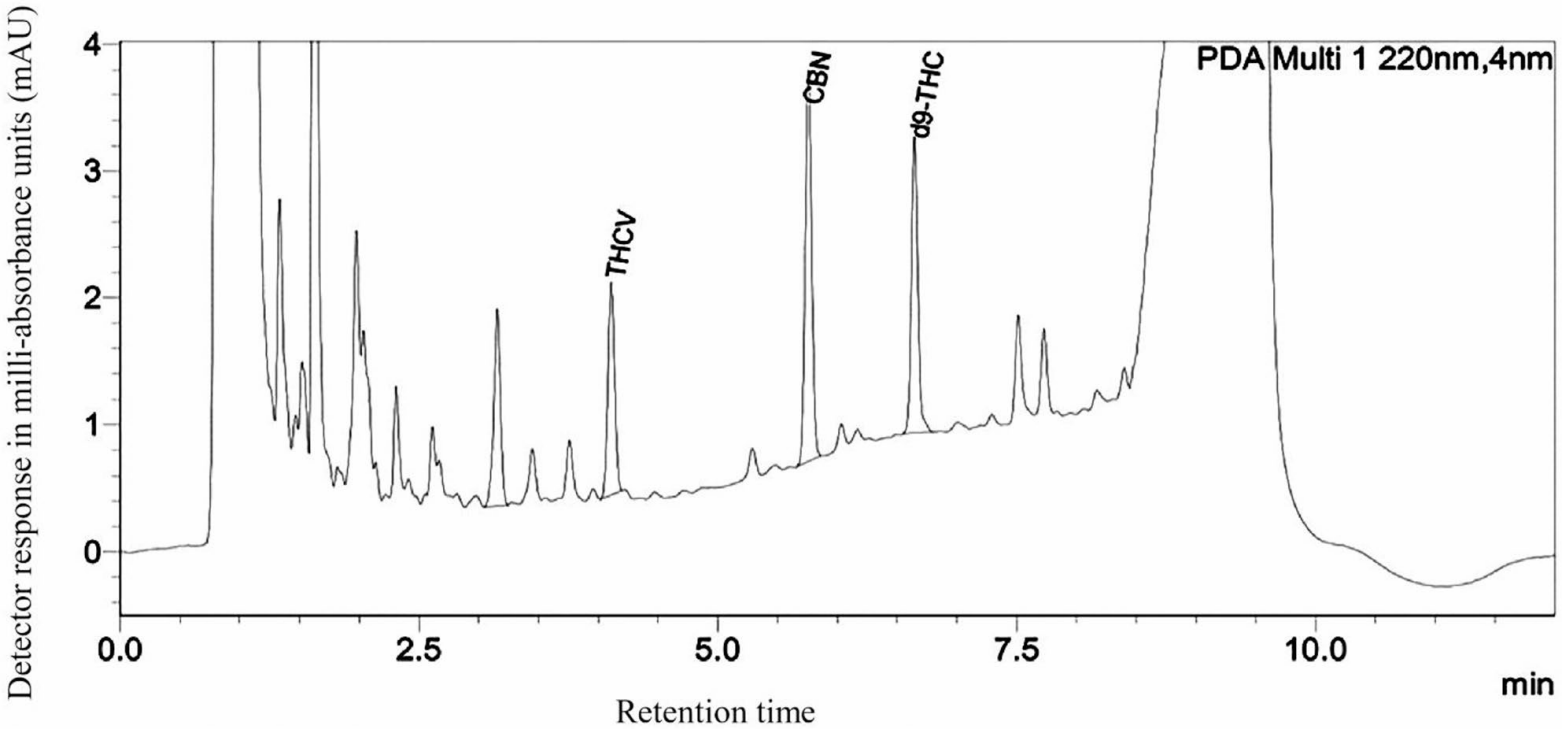
HPLC chromatogram of cannabinoids in the Anti-Pom-Leung Fever medicine. The x-axis represents retention time (min), and the y-axis represents detector response in milli-absorbance units (mAU)

High-performance liquid chromatography (HPLC) analysis (Fig. 2; Table 3) identified delta-9-tetrahydrocannabinol (Δ9-THC) as the predominant cannabinoid (6.645 mg/g), followed by cannabinol (CBN; 5.759 mg/g) and tetrahydrocannabivarin (THCV; 4.103 mg/g).

**Table 3:** Analysis of the cannabinoid content in the Anti-Pom-Leung Fever medicine formulation using High-Performance Liquid Chromatography (HPLC)

Compound	Amount (mg/g extract)
CBDV	ND
CBDA	ND
CBGA	ND
CBG	ND
CBD	ND
THCV	4.103 ± 0.005
CBN	5.759 ± 0.001
Δ^9^ -THC	6.645 ± 0.001
CBC	ND
THCA	ND

*Abbreviation in Table: CBDV* Cannabidivarin, *CBDA* Cannabidiolic Acid, *CBGA* Cannabigerolic Acid, *CBG* Cannabigerol, *CBD* Cannabidiol, *THCV* Tetrahydrocannabivarin, *CBN* Cannabinol, *Δ9-THC* Delta-9-tetrahydrocannabinol, *CBC* Cannabichromene, *THCA* Tetrahydrocannabinolic Acid, and *ND* Not Detected

### Safety assessments

Laboratory safety assessments demonstrated no statistically significant differences between the experimental and comparator groups after 28 days of treatment across a broad range of parameters (*p* > 0.05; Table 4). These included vital signs; hematological indices (WBC, HB, and PLT); pancreatic function (fasting blood glucose); renal function (creatinine); and lipid profiles, with all laboratory values remaining within normal clinical ranges. Similarly, liver function markers (AST, ALT, and ALP) and bilirubin levels showed no significant between-group differences and remained within reference limits throughout the study period (*p* > 0.05). Blood pressure and BMI also did not differ significantly between the two groups (*p* > 0.05).

**Table 4:** Clinical and laboratory safety parameters of the experimental and comparator groups before and after treatment

Parameter	Before treatment			After treatment		
	Experimental group	Comparator group	*p*-value	Experimental group	Comparator group	*p*-value
*Clinical parameters*						
BP systolic (mmHg)	122.02 ± 11.51	120.95 ± 10.80	0.665	120 ± 10.51	121.15 ± 9.89	0.613
BP diastolic (mmHg)	76.9 ± 7.92	73.68 ± 9.33	0.960	76 ± 11.26	74.54 ± 8.64	0.511
EKG change	5(12.2%)	8(19.5%)	0.265	6(14.6%)	8(19.5%)	0.775
*Laboratory parameters*						
CHO (mg/dL)	218.17 ± 47.85	233.73 ± 46.63	0.140	216.49 ± 40.64	223.22 ± 46.31	0.486
TG (mg/dL)	120.56 ± 77.35	124.68 ± 80.55	0.814	131.15 ± 93.23	122.63 ± 70.21	0.642
HDL (mg/dL)	57.22 ± 11.89	59.28 ± 14.66	0.490	55.49 ± 11.76	57.22 ± 13.37	0.535
LDL (mg/dL)	144.29 ± 58.00	153.05 ± 44.43	0.449	137.03 ± 36.28	139.55 ± 48.61	0.793
WBC (cells/uL)	6.48 × 10^3^ ± 1.46	6.59 × 10^3^ ± 1.86	0.785	6.09 × 10^3^ ± 1.13	6.36 × 10^3^ ± 1.79	0.412
HB (g/dL)	12.93 ± 1.22	12.95 ± 0.98	0.937	13.13 ± 1.24	12.96 ± 1.11	0.525
PLT (cells/uL)	261.34 × 10^3^ ± 55.43 × 10^3^	278.37 × 10^3^ ± 81.01 × 10^3^	0.271	255.61 × 10^3^ ± 49.30 × 10^3^	280.66 × 10^3^ ± 72.94 × 10^3^	0.073
AST (U/L)	22.22 ± 7.75	28.8 ± 13.93	0.815	23.90 ± 10.05	27.93 ± 36.42	0.957
ALT (U/L)	21.8 ± 13.13	22 ± 18.90	0.957	25.05 ± 23.05	25.93 ± 35.13	0.497
ALP (U/L)	67.95 ± 19.80	62.57 ± 19.83	0.223	68.10 ± 20.12	66.29 ± 27.15	0.733
Bilirubin (mg/dL)	0.54 ± 0.25	0.61 ± 0.50	0.397	0.60 ± 0.23	0.64 ± 0.38	0.621
FBS (mg/dL)	96.07 ± 13.03	97.37 ± 34.91	0.825	95.48 ± 12.79	95.27 ± 14.67	0.949
Cr (mg/dL)	0.76 ± 0.18	0.76 ± 0.20	0.973	0.75 ± 0.18	0.75 ± 0.19	0.895

*Abbreviations: BMI* body mass index, *BP* blood pressure, *EKG* change, presence of clinically significant electrocardiogram abnormalities, *CHO* cholesterol, *TG* triglycerides, *HDL* high-density lipoprotein, *LDL* low-density lipoprotein, *WBC* white blood cell count, *HB* hemoglobin, *PLT* platelet count, *AST* aspartate aminotransferase (formerly SGOT), *ALT* alanine aminotransferase (formerly SGPT), *ALP* alkaline phosphatase, *FBS* fasting blood sugar, *Cr* creatinine. Data are presented as mean ± standard deviation (SD) unless otherwise indicated. Intergroup comparisons at each time point were performed using the independent-samples t-test for continuous variables, while categorical variables were compared using the chi-square test. A *p* value < 0.05 was considered statistically significant

Clinical safety assessments further indicated no significant differences between groups. Blood pressure measurements remained comparable throughout the study period (*p* > 0.05). Cardiac monitoring by EKG revealed no significant differences between medication groups in heart rate or rhythm abnormalities (*p* > 0.05). Although minor EKG changes were observed post-treatment in 5 participants in the experimental group (mainly sinus bradycardia) and 8 participants in the comparator group (primarily conduction abnormalities), these findings were not statistically significant and did not lead to study withdrawal.

Minor adverse events observed in both treatment groups primarily included gastrointestinal irritation, dizziness, drowsiness, palpitations, and transient fever (Table 5). No statistically significant differences in the incidence of adverse events were observed between the groups (*p* > 0.05). All events were mild in severity, resolved spontaneously, and did not require medical intervention or result in treatment discontinuation.

**Table 5:** Comparison of the incidence of adverse events between the Anti-Pom-Leung Fever group and the Lorazepam group over the 4-week study period

Adverse Event	Anti-Pom-Leung Fever (*n* = 41)	Lorazepam (*n* = 41)	*p*-value
Gastric irritation	1 (2.4%)	1 (2.4%)	1.000
Dizziness	4 (9.8%)	1 (2.4%)	0.360
Drowsiness	1 (2.4%)	0 (0.0%)	1.000
Palpitations	1 (2.4%)	0 (0.0%)	1.000
Fever	0 (0.0%)	1 (2.4%)	1.000

The comparison of proportions between the two groups was performed using Fisher’s exact test due to the low frequency of reported events (expected counts ≤ 5). A *p* value > 0.05 was considered not statistically significant

### Sleep quality

**Fig. 3:**
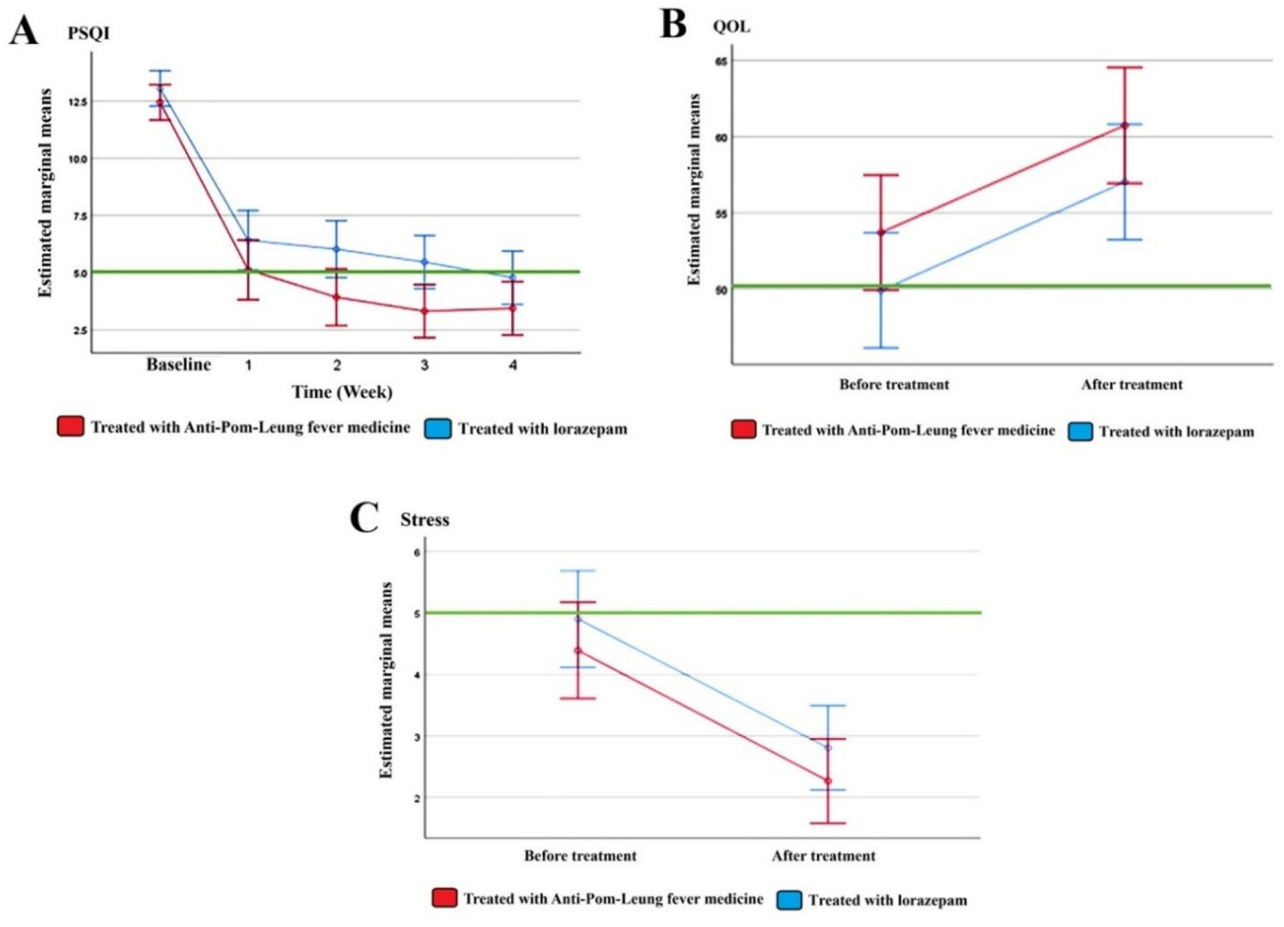
Changes in PSQI, quality of life, and stress scores over time. Profile plots of estimated marginal means (± 95% confidence intervals) for (**A**) Pittsburgh Sleep Quality Index (PSQI), (**B**) quality of life (QOL), and (**C**) stress scores across the study period. The experimental group (Anti-Pom-Leung Fever medicine) is shown in red, and the comparator group (lorazepam) is shown in blue

Sleep quality, as assessed by the PSQI, showed progressive improvement over the 4-week study period in both treatment and comparator groups. In the experimental group, mean PSQI scores decreased from 12.44 at baseline to 5.12 at week 1, 3.93 at week 2, 3.32 at week 3, and 3.44 at week 4 (Fig. 3A). A similar pattern was observed in the comparator group, with mean PSQI scores declining from 13.05 at baseline to 6.41 at week 1, 6.02 at week 2, 5.46 at week 3, and 4.78 at week 4. These findings indicate sustained improvement in sleep quality over time in both groups.

Repeated-measures ANOVA demonstrated a significant effect of time on PSQI scores (F = 217.71, *p* < 0.05, partial η² = 0.731; Table 6), indicating that sleep quality improved significantly over time. The time-by-treatment interaction was not statistically significant (*p* > 0.05), suggesting that the pattern of improvement over time was similar between the two groups. However, a small but statistically significant main effect of treatment group was observed (*p* < 0.05), indicating a modest overall difference in PSQI scores between the experimental and comparator groups across all time points.

**Table 6:** Results of repeated-measures ANOVA for PSQI, quality of life (QOL), and stress scores

Outcome	Effect	df	F	*p*-value	Partial η²
PSQI (Sleep Quality)	Time	2.42, 193.53†	217.71	< 0.001	0.731
	Time × Treatment	2.42, 193.53†	1.71	0.176	0.021
	Treatment group	1, 80	4.78	0.032	0.056
QOL (Quality of Life)	Time	1, 80	44.63	< 0.001	0.358
	Time × Treatment	1, 80	0.002	0.963	< 0.001
	Treatment group	1, 80	2.31	0.133	0.028
Stress	Time	1, 80	60.58	< 0.001	0.431
	Time × Treatment	1, 80	0.002	0.964	< 0.001
	Treatment group	1, 80	1.37	0.245	0.017

† Greenhouse–Geisser correction applied for PSQI due to violation of sphericity (Mauchly’s test *p* < 0.001). For QOL and Stress (two time points), sphericity was not violated

For the non-inferiority analysis, the primary endpoint at week 4 showed a mean PSQI score of 3.44 in the experimental group and 4.78 in the comparator group. The mean difference (experimental minus comparator) was − 1.34, with a 95% confidence interval ranging from − 2.99 to 0.31. Because the upper bound of the 95% confidence interval did not exceed the predefined non-inferiority margin of 2.1, non-inferiority of the experimental treatment compared with the comparator was demonstrated.

### Quality of Life

Quality of life improved over the 4-week study period in both the experimental and comparator groups. In the experimental group, the mean QOL score decreased from 4.39 at baseline to 2.27 at the post-intervention assessment (Fig. 3B). Similarly, in the comparator group, the mean QOL score decreased from 4.90 at baseline to 2.80 after the intervention, indicating an overall improvement in quality of life over time in both groups.

Repeated-measures analysis of variance demonstrated a significant main effect of time on QOL scores (F = 44.63, *p* < 0.05, partial η² = 0.358; Table 6), confirming a significant improvement in quality of life across the study period. The time-by-treatment interaction was not statistically significant (*p* > 0.05), indicating that the pattern of change over time did not differ between the experimental and comparator groups. In addition, the main effect of treatment group was not statistically significant (*p* > 0.05), suggesting no significant overall difference in QOL scores between the two groups across time points (Table 6).

### Stress

Stress levels, measured using the ST-5 scale, showed a clear reduction over the study period in both the experimental and comparator groups (Fig. 3C). In the experimental group, the mean ST-5 score decreased from 4.39 at baseline to 2.27 at the post-intervention assessment. A comparable reduction was observed in the comparator group, with mean ST-5 scores declining from 4.90 at baseline to 2.80 after the intervention, indicating an overall improvement in stress levels over time in both groups.

Repeated-measures analysis of variance revealed a significant main effect of time on ST-5 scores (F = 60.58, *p* < 0.05, partial η² = 0.431; Table 6), confirming a substantial decrease in stress levels throughout the study period. No significant time-by-treatment interaction was detected (*p* > 0.05), suggesting that the trajectory of stress reduction over time was similar between the experimental and comparator groups. Furthermore, the main effect of treatment group was not statistically significant (*p* > 0.05), indicating no significant overall difference in stress levels between the two groups across the study period (Table 6).

## Discussion

The present study demonstrates that the traditional Thai cannabis-based Anti-Pom-Leung Fever medicine is non-inferior to lorazepam with respect to the primary endpoint of sleep quality, as measured by the PSQI at week 4. This finding supports the clinical utility of the traditional formulation as an effective alternative to a standard benzodiazepine in the short-term management of chronic insomnia. Both treatment groups exhibited significant improvements in sleep quality over the four-week study period, and the absence of a significant time-by-treatment interaction indicates that the overall trajectory of improvement was comparable between the experimental and comparator groups.

High-performance liquid chromatography analysis identified delta-9-tetrahydrocannabinol (Δ9-THC) as the predominant cannabinoid in the Anti-Pom-Leung Fever formulation, followed by cannabinol (CBN) and tetrahydrocannabivarin (THCV). These phytocannabinoids have been reported to influence sleep regulation through modulation of central nervous system pathways, including CB1 receptor activation and interactions with orexin-mediated arousal systems (Berrendero et al. 2018). Although THCV is more commonly associated with metabolic regulation, its presence within a complex phytochemical matrix may contribute to an overall entourage effect that enhances sedative and anxiolytic properties and supports stabilization of the sleep-wake cycle (Russo 2011; D’Angelo and Steardo 2024). Accordingly, the observed clinical effects are biologically plausible but cannot be attributed to any single cannabinoid component.

Beyond cannabinoids, the Anti-Pom-Leung Fever medicine comprises a combination of traditional herbs, including red sandalwood, white sandalwood, neem leaf, and five-leaved chaste leaf, which have been historically associated with anti-inflammatory, anxiolytic, and sedative properties (Zheng and Qin 2011; Srinivasa Reddy and Neelima 2022). The inclusion of dry ginger, black pepper, and *Piper retrofractum* may further enhance tolerability and gastrointestinal absorption, while potentially mitigating adverse effects associated with isolated cannabinoid exposure (Russo 2011). The therapeutic effects observed in this study are therefore likely to reflect synergistic interactions among multiple bioactive constituents rather than a single pharmacological mechanism.

Improvements in PSQI scores observed in the experimental group are consistent with emerging clinical evidence supporting the role of cannabinoid-based therapies in insomnia management. (Lavender et al. 2023) reported that formulations containing combinations of THC and CBN may reduce sleep latency and improve subjective sleep quality, while potentially producing fewer residual next-day effects than conventional benzodiazepines. In the present study, participants receiving the Anti-Pom-Leung Fever medicine appeared to reach the PSQI threshold indicative of good sleep quality (PSQI ≤ 5) earlier than those receiving lorazepam; however, this observation should be interpreted cautiously, as formal time-to-event analyses were not performed and the overall pattern of improvement over time did not differ significantly between treatment groups.

Compared with previous studies, our study showed large reductions in PSQI scores, with decreases of 9.00 points in the experimental group and 8.27 points in the lorazepam group from baseline to week 4. These reductions are greater than those reported by the Institute of Geriatric Medicine (2008) for a behavioral self-help intervention in elderly patients, which showed a mean reduction of approximately 5.4 points. Although the study conducted by the Institute of Geriatric Medicine (2008) involved a small elderly cohort and a non-pharmacological intervention, it provides a contextual benchmark suggesting that the improvements observed in our trial exceed those typically achieved by behavioral interventions alone. In addition, when compared with prior studies evaluating cannabinoid-based interventions for sleep, the magnitude of PSQI improvement in our study appears larger. For example, (Ware et al. 2010) reported a PSQI reduction of approximately 3 points with nabilone, a synthetic cannabinoid, in patients with insomnia and fibromyalgia, while Shannon et al. (2019) observed a reduction of about 3.8 points with oral cannabidiol over three months. In contrast, our study demonstrated PSQI reductions of 9.00 points in the experimental group and 8.27 points in the lorazepam group over four weeks. These differences may partly reflect variations in study design, patient populations, baseline insomnia severity, and treatment duration. The cannabinoid studies involved heterogeneous populations and, in some cases, observational designs, whereas our study was a randomized controlled non-inferiority trial with an active comparator. Therefore, direct comparisons should be interpreted cautiously. Nevertheless, the magnitude of improvement observed in our study falls within or above the range reported for active pharmacological treatments for insomnia, supporting the clinical relevance of the observed effects.

A major limitation of this study is the absence of a placebo-controlled comparison group, as the non-inferiority design used an active comparator. This leaves open the theoretical possibility that some of the observed improvements could reflect placebo effects. However, evidence from placebo-controlled sleep trials suggests that placebo responses are typically modest. For example, a randomized double-blind placebo-controlled crossover trial of medicinal cannabis reported an improvement of approximately 2 points on the Insomnia Severity Index in the placebo group over two weeks (Ried et al. 2023). Similarly, in a randomized placebo-controlled study of cannabis oil that assessed sleep quality using the PSQI, placebo PSQI scores remained unchanged over four weeks (8.8 at baseline and 8.8 at week 4) (So-ngern et al. 2025). In contrast, the PSQI reductions observed in our study were substantially larger, reaching 8–9 points over four weeks. Although cross-study comparisons should be interpreted cautiously, the magnitude of improvement observed in our trial appears greater than that generally attributed to placebo responses, supporting the clinical relevance of the findings.

Safety findings of the Anti-Pom-Leung Fever medicine further support the clinical viability of the traditional formulation. No statistically significant between-group differences were observed in hepatic, renal, hematological, or metabolic laboratory parameters, and all values remained within clinically acceptable ranges. Cardiovascular monitoring demonstrated no significant differences between groups, and although minor electrocardiographic changes were observed in both arms, these events were mild, transient, and did not necessitate treatment discontinuation. The comparable incidence and severity of adverse events between groups suggest that the traditional formulation does not confer additional short-term safety risks relative to lorazepam.

In addition to sleep quality, both quality of life and stress scores improved significantly over time in both groups, with no significant time-by-treatment interactions or overall between-group differences. These findings suggest that improvements in sleep quality were accompanied by broader psychosocial benefits, although these secondary outcomes do not indicate superiority of one treatment over the other. Rather, they provide supportive evidence that the non-inferior sleep-related efficacy of the experimental treatment is not offset by negative effects on well-being or stress.

A key strength of this study lies in its randomized, double-blind, active-controlled design, which provides a robust framework for evaluating non-inferiority against an established standard therapy. The predefined non-inferiority margin and chemical characterization of the investigational product further strengthen the interpretability of the findings. Nevertheless, several limitations should be acknowledged. First, although per-protocol analysis is conservative and appropriate in non-inferiority trials, the exclusion of participants who did not complete the study may introduce attrition-related bias. Future analyses incorporating intention-to-treat populations would strengthen confidence in the generalizability of these findings. Second, the 28-day treatment period limits conclusions regarding long-term efficacy, tolerance, and dependence. Third, reliance on subjective sleep measures without objective assessments such as polysomnography or actigraphy precludes detailed evaluation of sleep architecture. Finally, the use of an indigenous Thai cannabis strain within a specific regulatory context may limit direct extrapolation to other formulations or populations.

## Conclusions

This Phase II trial demonstrates that the traditional Thai cannabis-based Anti-Pom-Leung Fever medicine is as effective and safe as lorazepam for the short-term treatment of chronic insomnia. The herbal formulation showed non-inferiority in improving subjective sleep quality while maintaining a favorable safety profile, with no significant adverse effects on hepatic, renal, or cardiac function. These findings support the integration of traditional cannabis-based medicine into evidence-based healthcare as a viable alternative to conventional benzodiazepines. Future research should prioritize long-term longitudinal studies and the optimization of concentrated extract formulations to enhance patient adherence and broaden clinical applicability.

## Data Availability

The raw data supporting the conclusions of this article will be made available by the authors on request.

## References

[CR1] Berrendero F, Flores A, Robledo P. When orexins meet cannabinoids: Bidirectional functional interactions. Biochem Pharmacol. 2018;157:43–50. 10.1016/j.bcp.2018. 08.040. 30171834 10.1016/j.bcp.2018.08.040

[CR2] Buysse DJ, Reynolds CF, Monk TH, Berman SR, Kupfer DJ. The Pittsburgh Sleep Quality Index: a new instrument for psychiatric practice and research. Psychiatry Res. 1989;28(2):193–213. 10.1016/0165-1781(89)90047-4. 2748771 10.1016/0165-1781(89)90047-4

[CR3] D’Angelo M, Steardo L. Cannabinoids and sleep: Exploring biological mechanisms and therapeutic potentials. Int J Mol Sci. 2024;25(7):3603. 10.3390/ijms25073603. 38612415 10.3390/ijms25073603 PMC11011314

[CR4] Department of Thai Traditional and Alternative Medicine, Ministry of Public Health; Thai Sleep Disorders Society; Royal College of Psychiatrists of Thailand; Royal College of Physicians of Thailand. Clinical recommendations for diagnosis and management of insomnia in Thailand for adults 2025. Bangkok: Ministry of Public Health. 2025. Available from: https://cimjournal.com/special-articles/sleep-6

[CR5] Department of Mental Health, Ministry of Public Health. Stress Test-5 (ST-5). Nonthaburi: Department of Mental Health. 2023. [Cited 2024 May 22]. Available from: https://dmh.go.th/test

[CR6] Department of Medical Sciences, Ministry of Public Health. Thai Herbal Pharmacopoeia 2021. Bangkok: Department of Medical Sciences. 2021. Available from: https://bdn.moph.go.th

[CR7] Ghiasi N, Bhansali RK, Marwaha R. Lorazepam. StatPearls. Treasure Island (FL): StatPearls Publishing; 2024. 30422485

[CR8] Herrera JG, Rolim LA, Honorato RS, Pimentel MF. Analysis of cannabinoids in medicinal cannabis products: A comprehensive review. J Braz Chem Soc. 2024;35(10):e–20240129. 10.21577/0103-5053.20240129.

[CR9] Institute of Geriatric Medicine, Department of Medical Services, Ministry of Public Health. Implementation of Self Help Group for Insomnia in the Elderly. Nonthaburi: Institute of Geriatric Medicine. 2008. [Cited 2026 Jan 31]. Available from: https://agingthai.dms.go.th/agingthai/wp-content/uploads/2020/07/book_8.pdf

[CR10] Jamparngernthaweesri K, Lumlerdkij N, Booranasubkajorn S. A retrospective study of Suk Sai Yad recipe uses in patients with insomnia. Siriraj Med Bull. 2023;16:119–30.

[CR11] Kamoltham T, Luangpirom N, Kuamsab N, Kummalue T, Chaiphongpachara T. Whole-genome sequencing and SNP analysis of Thai *Cannabis sativa* cultivar ‘Hang Kra Rog Phu Phan’ (Cannabaceae). Biodiversitas. 2025;26(10):4946–53. 10.13057/biodiv/d261010.

[CR12] Lavender I, McCartney D, Marshall N, Suraev A, Irwin C, D’Rozario AL, Gordon CJ, Saini B, Grunstein RR, Yee B, McGregor I, Hoyos CM. Cannabinol (CBN; 30 and 300 mg) effects on sleep and next-day function in insomnia disorder (‘CUPID’ study): protocol for a randomised, double-blind, placebo-controlled, cross-over, three-arm, proof-of-concept trial. BMJ Open. 2023;13(8):e071148. 10.1136/bmjopen-2022-071148. 37612115 10.1136/bmjopen-2022-071148 PMC10450062

[CR13] Leewongcharoen N. Utilization of the athens insomnia scale-Thai version (AIS-Thai) among Thai people. Thai J Neurol. 2024;40:52–8.

[CR14] Minkel J, Krystal AD. Optimizing the pharmacologic treatment of insomnia: Current status and future horizons. Sleep Med Clin. 2013;8(3):333–50. 10.1016/j.jsmc.2013. 06.002. 24015116 10.1016/j.jsmc.2013.06.002 PMC3763861

[CR15] Patwardhan B, Mashelkar RA. Traditional medicine-inspired approaches to drug discovery: can Ayurveda show the way forward? Drug Discov Today. 2009;14(15–16):804–11. 10.1016/j.drudis.2009.05.009. Epub 2009 May 27. PMID: 19477288. 19477288 10.1016/j.drudis.2009.05.009

[CR16] Phattharayuttawat S, Ngamthipwatthana T, Pitiyawaranun B. The development of the pictorial Thai quality of life. J Med Assoc Thai. 2005;88:1605–18. 16471109

[CR17] Phraya Phitsanuphrasatwet. The Manual of Royal Thai Traditional Medicine. Vol. 1. Bangkok: Phraya Phitsanuphrasatwet; 1909.

[CR18] Ried K, Tamanna T, Matthews S, Sali A. Medicinal cannabis improves sleep in adults with insomnia: A randomised double-blind placebo-controlled crossover study. J Sleep Res. 2023;32(3):e13793. 36539991 10.1111/jsr.13793

[CR19] Roth T. Insomnia: definition, prevalence, etiology, and consequences. J Clin Sleep Med. 2007;3:3–6. PMC1978319 17824495

[CR20] Russo EB, Taming THC. Potential cannabis synergy and phytocannabinoid-terpenoid entourage effects. Br J Pharmacol. 2011;163(7):1344–64. 10.1111/j.1476-5381.2011.01238.x. 21749363 10.1111/j.1476-5381.2011.01238.x PMC3165946

[CR21] Shannon S, Lewis N, Lee H, Hughes S. Cannabidiol in anxiety and sleep: a large case series. Perm J. 2019;23. 10.7812/TPP/18-041 PMC6326553 30624194

[CR22] So-ngern A, Sripanichkulchai B, Mahakkanukrauh A, Suwannaroj S, Pongkulkiat P, Onchan T, Kanokmedhakul S, Foocharoen C. Efficacy of Cannabis Oil in Improving Subjective Sleep Quality in Systemic Sclerosis: A Prospective Placebo-Controlled Study. Life. 2025;15(5):727. 40430155 10.3390/life15050727 PMC12113143

[CR23] Srinivasa Reddy IV, Neelima P. Neem (*Azadirachta indica*): A review on medicinal Kalpavriksha. Int J Econ Plants. 2022;9(1):59–63. 10.23910/2/2021.0437d.

[CR24] United States Pharmacopeial Convention. <2040 > Disintegration and dissolution of dietary supplements. United States Pharmacopeia and National Formulary (USP 40-NF 35). Rockville, MD: United States Pharmacopeial Convention; 2017a.

[CR25] United States Pharmacopeial Convention. <2091 > Weight variation of dietary supplements. United States Pharmacopeia and National Formulary (USP 40-NF 35). Rockville, MD: United States Pharmacopeial Convention; 2017b.

[CR26] Walsh JH, Maddison KJ, Rankin T, Murray K, McArdle N, Ree MJ, Hillman DR, Eastwood PR. Treating insomnia symptoms with medicinal cannabis: A randomized, crossover trial of the efficacy of a cannabinoid medicine compared with placebo. Sleep. 2021;44:1–8. 10.1093/sleep/zsab149 PMC8598183 34115851

[CR27] Ware MA, Fitzcharles M-A, Joseph L, Shir Y. The effects of nabilone on sleep in fibromyalgia: results of a randomized controlled trial. Anesth Analg. 2010;110(2):604e10. 20007734 10.1213/ANE.0b013e3181c76f70

[CR28] Zheng CJ, Qin LP. Five-leaved chaste tree (Vitex negundo) seeds and antinociceptive effects. In: Nuts and seeds in health and disease prevention. 2011;479–86. 10.1016/B978-0-12-375688-6.10057-X

